# Clinical Characteristics of Patients With Coronavirus Disease 2019 (COVID-19) Receiving Emergency Medical Services in King County, Washington

**DOI:** 10.1001/jamanetworkopen.2020.14549

**Published:** 2020-07-08

**Authors:** Betty Y. Yang, Leslie M. Barnard, Jamie M. Emert, Christopher Drucker, Leilani Schwarcz, Catherine R. Counts, David L. Murphy, Sally Guan, Kosuke Kume, Karen Rodriquez, Tracie Jacinto, Susanne May, Michael R. Sayre, Thomas Rea

**Affiliations:** 1 Department of Emergency Medicine, University of Washington, Seattle; 2 Division of Emergency Medical Services Public Health–Seattle and King County, Seattle, Washington; 3 Department of Biostatistics, University of Washington, Seattle; 4 Seattle Fire Department, Seattle, Washington; 5 Department of Medicine, University of Washington, Seattle

## Abstract

**Importance:**

The ability to identify patients with coronavirus disease 2019 (COVID-19) in the prehospital emergency setting could inform strategies for infection control and use of personal protective equipment. However, little is known about the presentation of patients with COVID-19 requiring emergency care, particularly those who used 911 emergency medical services (EMS).

**Objective:**

To describe patient characteristics and prehospital presentation of patients with COVID-19 cared for by EMS.

**Design, Setting, and Participants:**

This retrospective cohort study included 124 patients who required 911 EMS care for COVID-19 in King County, Washington, a large metropolitan region covering 2300 square miles with 2.2 million residents in urban, suburban, and rural areas, between February 1, 2020, and March 18, 2020.

**Exposures:**

COVID-19 was diagnosed by reverse transcription–polymerase chain reaction detection of severe acute respiratory syndrome coronavirus 2 from nasopharyngeal swabs. Test results were available a median (interquartile range) of 5 (3-9) days after the EMS encounter.

**Main Outcomes and Measures:**

Prevalence of clinical characteristics, symptoms, examination signs, and EMS impression and care.

**Results:**

Of the 775 confirmed COVID-19 cases in King County, EMS responded to 124 (16.0%), with a total of 147 unique 911 encounters. The mean (SD) age was 75.7 (13.2) years, 66 patients (53.2%) were women, 47 patients (37.9%) had 3 or more chronic health conditions, and 57 patients (46.0%) resided in a long-term care facility. Based on EMS evaluation, 43 of 147 encounters (29.3%) had no symptoms of fever, cough, or shortness of breath. Based on individual examination findings, fever, tachypnea, or hypoxia were only present in a limited portion of cases, as follows: 43 of 84 encounters (51.2%), 42 of 131 (32.1%), and 60 of 112 (53.6%), respectively. Advanced care was typically not required, although in 24 encounters (16.3%), patients received care associated with aerosol-generating procedures. As of June 1, 2020, mortality among the study cohort was 52.4% (65 patients).

**Conclusions and Relevance:**

The findings of this cohort study suggest that screening based on conventional COVID-19 symptoms or corresponding examination findings of febrile respiratory illness may not possess the necessary sensitivity for early diagnostic suspicion, at least in the prehospital emergency setting. The findings have potential implications for early identification of COVID-19 and effective strategies to mitigate infectious risk during emergency care.

## Key Points

**Question:**

What is the clinical presentation to emergency medical services among persons with coronavirus disease 2019 (COVID-19)?

**Findings:**

This cohort study of 124 patients with COVID-19 revealed that most patients with COVID-19 presenting to emergency medical services were older and had multiple chronic health conditions. Initial concern, symptoms, and examination findings were heterogeneous and not consistently characterized as febrile respiratory illness.

**Meaning:**

The findings of this study suggest that the conventional description of febrile respiratory illness may not adequately identify COVID-19 in the prehospital emergency setting.

## Teaser

This cohort study describes patient characteristics and prehospital presentation of patients with coronavirus disease 2019 (COVID-19) cared for by emergency medical services (EMS) in King County, Washington.

## Introduction

The severe acute respiratory syndrome coronavirus 2 (SARS-CoV-2) pandemic was first reported in Hubei Province, China, in December 2019.^1,2^ The initial US case of coronavirus disease 2019 (COVID-19) was reported on January 20, 2020, in Washington state.^3^ The virus spread undetected until February 28, when it was identified in patients hospitalized in Kirkland, Washington.^4^ Subsequently, lab-confirmed cases of COVID-19 increased exponentially in King County, Washington, and other parts of the United States.

Although the clinical profile of patients has been reported,^5,6,7,8,9^ little is known regarding the presentation of patients with COVID-19 requiring emergency care and in particular about those who required 911 emergency medical services (EMS). EMS, with a US workforce of nearly half a million persons, provides critical access to the health system for patients with the most severe illness. EMS professionals are on the front line of health emergencies, responding urgently with incomplete information, to provide care in heterogeneous and sometimes uncontrolled circumstances. In this study, we describe the prehospital presentation and care of persons who required 911 EMS response and were ultimately diagnosed with COVID-19 to provide actionable insights to help to inform best practice.

## Methods

### Study Design, Setting, and Population

The study is a retrospective cohort investigation of patients with lab-confirmed COVID-19 in Seattle and greater King County, Washington, who required 911 EMS response from February 1, 2020, to March 18, 2020. The investigation was designed and reported with consideration of the Strengthening the Reporting of Observational Studies in Epidemiology (STROBE) reporting guideline.^10^ The study was approved by the University of Washington institutional review board. Because the investigation was considered minimal risk, the requirement for consent was waived.

COVID-19 was diagnosed by real-time reverse transcription–polymerase chain reaction (RT-PCR) detection of SARS-CoV-2 from nasopharyngeal swabs. Test results were available a median (interquartile range) of 5 (3-9) days after the EMS encounter.

King County is a large metropolitan region, covering 2300 square miles, with 2.2 million residents in urban, suburban, and rural areas. A total of 4 emergency communication centers provide 911 medical dispatch. The primary 911 medical response in King County is 2 tiered. The first tier is provided by firefighter emergency medical technicians. The second-tier response comprises paramedics, who are dispatched in cases of more severe illness. There are 28 first-tier fire departments and 5 overarching second-tier paramedic agencies that collectively provide primary emergency response to all 911 medical calls.

EMS is administered by Public Health–Seattle and King County, enabling direct engagement between EMS and Public Health to undertake disease surveillance. To identify patients with COVID-19 evaluated by EMS, we linked local and state COVID-19 surveillance systems with EMS electronic medical records using name, date of birth, and incident address.

### Data Sources and Abstraction

King County EMS maintains an electronic record of each EMS response. The current investigation used a uniform data abstraction form to review the narrative and formatted data fields of the dispatch and EMS records to assess patient characteristics (ie, chronic health conditions, symptoms, and examinations), call circumstances, and EMS care. On March 6, the electronic medical record incorporated the diagnosis of COVID-19, suspected or known. We also reviewed the narrative to assess noted and suspected COVID-19.

### Statistical Analysis

We report the distribution of characteristics overall and stratified by residential status (ie, long-term health care facility vs other residence). To compare characteristics according to residential status, we used descriptive statistics, the χ^2^ and Fisher exact tests for categorical variables, and *t* and Wilcoxon tests for continuous variables. All analyses were conducted on SPSS statistical software version 24 (IBM Corp). A *P* ≤ .05 was considered statistically significant, and all tests were 2-tailed.

## Results

From February 1, 2020, to March 18, 2020, there were 775 patients with lab-confirmed COVID-19 in King County. Of these, EMS responded to 124 patients (16.0%) with a total of 147 unique 911 encounters. A total of 66 patients (53.2%) were women, and the mean (SD) age was 75.7 (13.2) years (Table 1). A total of 56 patients (46.0%) were residents in long-term care facilities, and 47 (37.9%) had 3 or more chronic health conditions. The most common health conditions were hypertension (44 [35.4%]), cardiac disease (41 [33.1%]), lung disease (26 [21.0%]), diabetes (25 [20.2%]), and dementia (23 [18.5%]). Only 5 patients (4.0%) had no reported chronic health conditions, whereas health history was unknown for 14 (11.3%).

**Table 1.** Characteristics of Patients With Coronavirus Disease 2019 With 911 Emergency Medical Services Encounters

Characteristic	No. (%)			*P* value
	All patients (N = 124)	Residence in long-term care facility (n = 56)	Residence other than long-term care facility (n = 68)	
Age, mean (SD), y	75.7 (13.2)	80.7 (9.7)	71.4 (14.3)	<.001
Women	66 (53.2)	30 (53.6)	36 (52.9)	.72
Chronic health conditions				
None reported or missing	19 (15.3)	6 (10.7)	13 (19.1)	.08
1	21 (16.9)	7 (12.5)	14 (20.6)	
2	37 (29.8)	15 (26.8)	22 (32.4)	
≥3	47 (37.9)	28 (50.0)	19 (27.9)	
Individual health conditions				
Hypertension	44 (35.5)	20 (35.7)	24 (35.3)	.99
Any cardiac disease	41 (33.1)	20 (35.7)	21 (30.9)	.70
Cardiomyopathy	21 (16.9)	15 (26.8)	6 (8.8)	.02
Atrial fibrillation or other arrhythmias	16 (12.9)	11 (19.6)	5 (7.4)	.06
Any lung disease^a^	26 (21.0)	15 (26.8)	11 (16.2)	.19
Diabetes	25 (20.2)	9 (16.1)	16 (23.5)	.37
Dementia	23 (18.5)	16 (28.6)	7 (10.3)	.01
Neurologic or other	12 (9.7)	7 (12.5)	5 (7.4)	.55
Stroke or TIA	11 (8.9)	5 (8.9)	6 (8.8)	.99
Kidney disease or dialysis	7 (5.6)	6 (10.7)	1 (1.5)	.04
Cancer	5 (4.0)	4 (7.1)	1 (1.5)	.17
Immunocompromised	4 (3.2)	1 (1.8)	3 (4.4)	.63
Other^b^	15 (12.1)	8 (14.3)	7 (10.3)	.99
Recent history of pneumonia	18 (14.5)	10 (17.9)	8 (11.8)	.60
Mortality^c^	65 (52.4)	41 (73.2)	24 (35.3)	<.001

Abbreviation: TIA, transient ischemic attack.

^a^ Lung disease included chronic obstructive pulmonary disease (13 patients), asthma (7 patients), chronic bronchitis (1 patient), sarcoidosis (1 patient), and other (4 patients).

^b^ Other health conditions included thromboembolic disease (10 patients), affective disorders (4 patients), and liver disease (1 patient).

^c^ Mortality data were available through June 1, 2020.

The most common initial dispatch codes were for illness of unknown origin (41 encounters [27.9%]), difficulty breathing (37 [25.2%]), trauma (22 [15.0%]), and infectious disease (19 [12.9%]) (Table 2). In 91 dispatch assessments (61.9%), patients did not describe any of these symptoms. The most frequent symptoms reported by EMS documentation were fever (68 [46.2%]), followed by shortness of breath (64 [43.5%]), fatigue (59 [40.1%]), cough (43 [29.3%]), and altered mental status (41 [27.9%]). Based on EMS evaluation, patients in 43 encounters (29.3%) had no symptoms of fever, cough, or shortness of breath. Individual examination findings of fever, tachypnea, or hypoxia were only present in 43 of 84 encounters (51.2%), 42 of 131 encounters (32.1%), and 60 of 112 encounters (53.6%), respectively. Gastrointestinal symptoms were noted, including nausea and/or vomiting (14 encounters [9.5%]) and diarrhea (9 encounters [6.1%]). Decreased level of consciousness by Glasgow Coma Scale was present in 29 of 108 encounters (26.9%), and hypotension at presentation was observed in 16 of 134 encounters (11.9%).

**Table 2.** Characteristics of EMS Encounters With Patients With COVID-19

Characteristic	All encounters (N = 147)	Encounter at long-term care facility (n = 63)	Encounter not at long-term care facility (n = 84)	*P* value
Location of presentation				
Home	70 (47.6)	NA	70 (83.3)	NA
Facility				
Long-term care	63 (42.9)	63 (100)	NA	
Skilled nursing	51 (34.7)	51 (81.0)	NA	
Assisted living	12 (8.2)	12 (19.0)	NA	
Outpatient	11 (7.5)	NA	11 (13.1)	
Public or street	3 (2.0)	NA	3 (3.6)	
Initial dispatch code				
Illness of unknown origin	41 (27.9)	17 (27.0)	24 (28.6)	.14
Difficulty breathing	37 (25.2)	15 (23.8)	22 (26.2)	
Trauma	22 (15.0)	12 (19.0)	10 (11.9)	
Infectious disease	19 (12.9)	7 (11.1)	12 (14.3)	
Cardiac	14 (9.5)	5 (7.9)	9 (10.7)	
Bleeding or pain, nontraumatic	8 (5.4)	6 (9.5)	2 (2.4)	
Stroke or headache	6 (4.1)	0	6 (7.1)	
Documented symptoms				
Fever, cough, or shortness of breath	104 (70.7)	41 (65.1)	63 (75.0)	.20
Cough	43 (29.3)	9 (14.3)	34 (40.5)	.001
Fever	68 (46.3)	28 (44.4)	40 (47.6)	.74
Shortness of breath	64 (43.5)	28 (44.4)	36 (42.9)	.87
Fatigue	59 (40.1)	16 (25.4)	43 (51.2)	.002
Altered mental status	41 (27.9)	21 (33.3)	20 (23.8)	.27
Nausea or vomiting	14 (9.5)	1 (1.6)	13 (15.5)	.004
Diarrhea	9 (6.1)	1 (1.6)	8 (9.5)	.08
Headache	4 (2.7)	1 (1.6)	3 (3.6)	.64
Sore throat	3 (2.0)	0	3 (3.6)	.26
Muscle aches or joint pain	1 (0.7)	0	1 (1.2)	.99
Other^a^	5 (3.4)	4 (6.3)	1 (1.2)	.17
Temperature, mean (SD), °C^b^	37.9 (1.1)	38.1 (1.0)	37.7 (1.2)	.16
Abnormal initial vital sign results, No./total No. (%)				
Heart rate ≥100 bpm	47/137 (34.3)	19/62 (30.6)	28/75 (37.3)	.47
Body temperature ≥38 °C	43/84 (51.2)	24/42 (57.1)	19/42 (45.2)	.38
Respiratory rate ≥24	42/131 (32.1)	27/58 (46.6)	15/73 (20.5)	.002
Oxygenation saturation ≤92%	60/112 (53.6)	30/55 (54.5)	30/57 (52.6)	.85
Glasgow Coma Scale score <15	29/108 (26.9)	22/53 (41.5)	7/55 (12.7)	<.001
Systolic blood pressure ≤90 mm Hg	16/134 (11.9)	8/60 (13.3)	8/74 (10.8)	.79
Emergency medical services primary impression				
Flu-like symptoms	36 (24.5)	15 (23.8)	21 (25)	.40
Respiratory	30 (20.4)	12 (19.0)	18 (21.4)	
Weakness	19 (12.9)	5 (7.9)	14 (16.7)	
Injury or pain	14 (9.5)	9 (14.3)	5 (6)	
COVID-19^c^	12 (8.2)	6 (9.5)	6 (7.1)	
Altered mental status	8 ](5.4)	4 (6.3)	4 (4.8)	
Cardiac	7 (4.8)	2 (3.2)	5 (6.0)	
Other^d^	21 (14.3)	10 (15.9)	11 (13.1)	
EMS documented COVID-19				
Lab-confirmed COVID	10 (6.8)	4 (6.3)	6 (7.1)	.63
COVID-19 suspected by EMS	64 (43.5)	25 (39.7)	39 (46.4)	
No mention of COVID-19	73 (49.7)	34 (54.0)	39 (46.4)	
EMS care provided				
Oxygenation and ventilation support	49 (33.3)	27 (42.9)	22 (26.2)	.05
Highest level of support				
Nasal cannula or simple face mask	26 (17.7)	12 (19)	14 (16.7)	.07
Nonrebreather mask	19 (12.9)	11 (17.5)	8 (9.5)	
CPAP or BVM	2 (1.4)	2 (3.2)	0	
Intubation	2 (1.4)	2 (3.2)	0	
Intravenous fluid	16 (10.9)	9 (14.3)	7 (8.3)	.30
Nebulizer therapy	3 (2)	2 (3.2)	1 (1.2)	.58
Medication	7 (4.8)	4 (6.3)	3 (3.6)	.46
CPR	1 (0.7)	0	1 (1.2)	.43
Aerosol-generating procedures^e^	24 (16.3)	16 (25.4)	8 (9.5)	.01
Disposition				
Not transported	26 (17.7)	7 (11.1)	19 (22.6)	.008
BLS transport, fire and private ambulance	93 (63.3)	44 (69.8)	49 (58.3)	
ALS transport	23 (15.6)	12 (19.0)	11 (13.1)	
Transported by private vehicle	5 (3.4)	0	5 (6.0)	

Abbreviations: ALS, advanced life support; BLS, basic life support; bpm, beats per minute; BVM, bag valve mask; COVID-19, coronavirus disease 2019; CPAP, continuous positive airway pressure; CPR, cardiopulmonary resuscitation; EMS, emergency medical services; NA, not applicable.

^a^ Other symptoms included pain (3 patients), bleeding (1 patient), and vertigo (1 patient).

^b^ Body temperature measurements were available for 84 encounters.

^c^ COVID-19 impression became available to EMS on March 6, 2020.

^d^ Other primary impressions included no injury or illness noted (10 patients), vaginal hemorrhage (1 patient), skin infection (1 patient), seizure (1 patient), obvious death (1 patient), nausea (2 patients), gastrointestinal hemorrhage (1 patient), epistaxis (1 patient) and dehydration (1 patient), assist (1 patient), and urinary tract infection (1 patient).

^e^ Aerosol-generating procedures included CPAP, BVM, nebulizer therapy, nonrebreather mask, intubation, and CPR.

The primary EMS impression of encounters was flu-like symptoms (36 [24.5%]) or respiratory distress (30 [20.4%]), and 74 encounters (50.3%) noted COVID-19 in their report or impression. Advanced care was typically not required, although in 24 encounters (16.3%), patients received care associated with aerosol-generating procedures (Table 2). A total of 49 encounters (33.3%) included oxygen therapy and/or ventilation support.

Compared with those who did not reside in a long-term care facility, patients from a long-term facility were older (mean [SD] age, 80.7 [9.7] years vs 71.4 [14.3] years; *P* < .001), presented with a Glasgow Coma Scale score of less than 15 (22 of 53 encounters [41.5%] vs 7 of 55 [12.7%]; *P* < .001), and more often manifested tachypnea (27 of 58 encounters [46.6%] vs 15 of 73 encounters [20.5%]; *P* = .002) (Table 1 and Table 2). Overall mortality among the cohort was 52.4% (65 of 124) as of June 1, 2020. Mortality was greater among those residing in a long-term care facility (41 of 56 [73.2%] vs 24 of 68 [35.3%]; *P* < .001) (Table 1).

## Discussion

In this cohort investigation, EMS was involved in 124 of 775 cases of COVID-19 (16.0%) during the first 20 days since the initial diagnosis in King County, Washington. The cohort was characterized by substantial chronic health comorbidities, 46.0% of patients resided in long-term care facilities, and 52.4% died by June 1, 2020. These observations are consistent with reports demonstrating older persons and those with comorbidities have the highest risk of mortality related to COVID-19 and so could be expected to have more severe illness and require EMS and emergency care.^5,6,11^

Of the 147 EMS encounters, 91 dispatch assessments (61.9%) and 43 EMS evaluations (29.3%) for patients with COVID-19 did not present with symptoms of fever, respiratory difficulty, or cough. Instead there was a range of primary symptoms, including chest pain, altered mental status, weakness, and minor injury or pain, often resulting from a fall. Similarly, approximately half of patients exhibited individual signs of measured fever (43 of 84 [51.2%]) or hypoxia (60 of 112 [53.6%]), and fewer than one-third experienced tachypnea (42 of 131 [32.1%]). One might consider that skilled nursing status could be a strong confounder in presentation. Although there was some evidence of presentation difference based on residence status, nonspecific symptoms and signs were prevalent among those residing outside long-term care facilities. Moreover, this heterogeneity was reflected in the EMS impression. This observation suggests that screening based on conventional febrile respiratory illness symptoms of COVID-19 or corresponding examination findings may not possess the necessary sensitivity for early diagnostic suspicion, at least in the prehospital emergency circumstance.

One-third of encounters (49 of 147 [33.3%]) required oxygen therapy and/or ventilation support, with 24 encounters (16.3%) including an aerosol-generating treatment that may increase risk of transmission.^12,13,14^ However, there is little information regarding occupational risk for EMS during the current COVID-19 pandemic, although there are lay reports suggesting that EMS professionals may be at high risk.^15^ Rigorous evaluation is required to define occupational risk and determine what strategies effectively mitigate risk.^16^

### Limitations

This study has limitations. We relied on dispatch and EMS reports to ascertain clinical information, resulting in some missingness and potential misclassification. For example, the prevalence of chronic health conditions documented by EMS is likely an underestimate, and the comorbidities overall are likely even more prevalent. Nonetheless, EMS ascertainment of comorbidity appears to be a meaningful strategy to assess health status.^17,18^ The study evaluated EMS involvement with confirmed COVID-19 cases. There may be EMS encounters in which a patient had COVID-19 but was not tested. However, patients requiring EMS likely have more severe disease and thus may be prioritized for testing.^19^ The study population was derived from a single, large EMS system, and the sample size was modest. Hence, we are cautious regarding generalizability and about drawing definitive inference in comparing characteristics, eg, according to residential status. Nonetheless, the ability to link EMS and surveillance records makes for a valuable public health investigative tool that can help inform clinical strategies for emergency care during the pandemic.

## Conclusions

In this high-risk cohort involving EMS response, symptoms and signs of COVID-19 were heterogeneous, suggesting a need to consider COVID-19 in some cases in which febrile respiratory illness is not prominent, at least in the emergency setting among patients who are older and have chronic comorbidities. In a subset, EMS provided interventions that may be associated with higher risk of transmission. Collectively, the findings have potential implications for early identification of COVID-19 and effective strategies to mitigate infectious risk during emergency care.
